# Early Life Physical Activity Patterns and Its Survival to Adult Activity Levels: The Longitudinal ABIS Study

**DOI:** 10.1186/s40798-025-00934-6

**Published:** 2025-11-21

**Authors:** Noman Sohail, Johnny Ludvigsson

**Affiliations:** 1 Division of Pediatrics, Department of Biomedical and Clinical Science, Linköping University, 58252 Linköping, Sweden; 2 Crown Princess Victoria Children’s Hospital, Region Östegötland, 58252 Linköping, Sweden

**Keywords:** ABIS, Physical activity, Screen time, Environmental factors, Health behavior

## Abstract

**Background and Aims:**

The impact of physical activity during early-life is significant on long-term health outcomes. The aim of this study was to determine what factors contribute to the continuity or change in activity behaviors over time.

**Methods:**

Out of 21,700 children born on Oct 1st, 1997 to Oct 1st, 1999, 17,055 (78.6%) were included in ABIS (All Babies in southeast Sweden) of whom 16,415 participants were included in this longitudinal prospective population-based birth cohort. Logistic regression was conducted to assess associations between the activity score and independent variables, with results presented as odds ratios, 95% confidence intervals, *p* values, and correlation coefficients (*r* values).

**Results:**

At age 3, high physical activity (Q2) was correlated to: living in a house (*r* = 0.881, *p* = 0.003) with both parents (*r* = 0.833, *p* < 0.001), ≥1 siblings (*r* = 0.876, *p* < 0.001), having a dog (*r* = 0.773, *p* < 0.001), high parental education (*r* = 0.817, *p* < 0.001) and parents working part time (0–50%) (*r* = 0.496, *p* < 0.001). These factors were persistent at age 5, 8, 10–12 years. Participation in sports had strong correlation with Q2 from age 8- to 17–23-years (*r* = 0.763, *p* < 0.001). Less physical activity (Q1) was correlated to: at age 3 parental less education (*r* = 0.816, *p* < 0.001) with full time work (*r* = 0.816, *p* < 0.001), no siblings (*r* = 0.835, *p* = 0.004), child lives in split custody (*r* = 0.736, *p* < 0.001), and mother smoking (*r* = 0.789, *p* = 0.032). These patterns seemed persistent at age 5, 8, 10–12 years with Q1. Lower parental smoking was associated to higher physical activity in children.

**Conclusion:**

The study identifies key factors affecting children and adolescents’ physical activity, providing insights for targeted interventions.

**Graphical Abstract:**

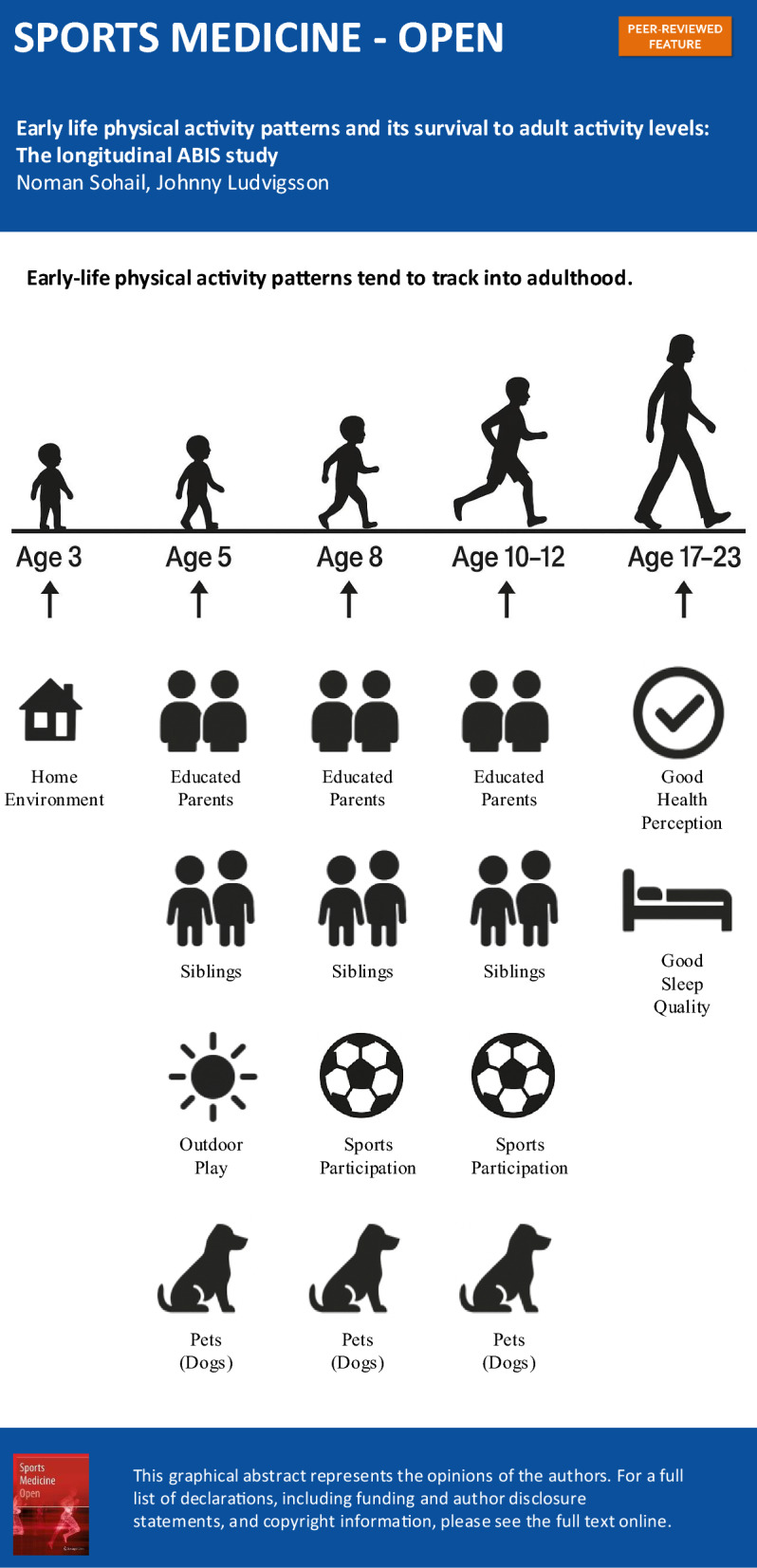

**Supplementary Information:**

The online version contains supplementary material available at 10.1186/s40798-025-00934-6.

## Key Points

- Early-life physical activity patterns tend to track into adulthood.
- Children who were medium physically active in childhood became more active after age 8.
- Sedentary behavior of children improved based on factors like parental education, parental part time job patterns, and organized physical activities for a child.

**Supplementary Information:**

The online version contains supplementary material available at 10.1186/s40798-025-00934-6.

## Introduction

Physical activity is a fundamental component of human health, influencing physical, mental, and social well-being across the lifespan [1, 2]. Establishing healthy activity patterns early in life is especially important, as these behaviors often track into adolescence and adulthood, with long-term implications for cardiovascular fitness, metabolic health, and overall quality of life [3, 4]. The early years of life represent a critical period for the development of these habits [5], and research suggests that children who regularly engage in physical activity are more likely to maintain active lifestyles later in life compared to their less active peers [3, 6]. While the continuity of physical activity has been well documented [7, 8], less is known about the environmental, familial, and behavioral factors that support or hinder these long-term behavioral patterns. Understanding not only whether physical activity patterns persists, but also why they persist or decline is essential for shaping effective strategies to promote lifelong physical activity and reduce chronic disease risk.

A range of factors influence children’s activity patterns [9–13], for example: parental education and employment play a critical role, as highly educated parents may place greater value on health-promoting behaviors and have more resources to support extracurricular activities. Family structure, including whether a child lives with both parents or in split custody, can affect stability and routine, which are important for activity maintenance. The presence of siblings, pets (e.g., dogs), and housing type (villas vs. apartments) are environmental influences that can facilitate physical movement. Additionally, factors like parental smoking, screen time, sleep quality, and participation in organized sports have all been associated with children’s activity behaviors and may contribute to changes as children transition to adulthood. Moreover, understanding how these shifts interact with early-life behaviors provides a more dynamic picture of how physical activity patterns evolve over time.

The aim of this study was to explore the continuity and change of physical activity patterns from early childhood into young adulthood specifically: (1) To what extent do early-life physical activity patterns persist into adulthood?, (2) What environmental and familial factors in early life are associated with high or low physical activity in adulthood? and (3) What changes in contextual factors (e.g., family, health, lifestyle) are linked to increases or decreases in activity levels over time? Thus, is it possible to facilitate in some way so that children who start physically active really remain physically active, or persuade those with a sedentary lifestyle to become more active? Significantly, this study offers a useful insight that can help create programs to encourage lifelong physical activity and reduce sedentary behavior in children.

## Methods

The data used in this study are from the longitudinal prospective population-based birth cohort study ABIS (All Babies in South-east Sweden). All 21,700 mothers who gave birth to a child between 1st of October 1997 and 1st of October 1999 were asked to participate in the ABIS study and 17,055 (78.6%) did so after providing their informed consent, with useful questionnaires obtained from 16,415 (75.7%). ABIS was designed to identify environmental and genetic factors associated with development of immune-mediated diseases, especially type 1 diabetes and other autoimmune diseases.

Questionnaires were answered by parents at the birth of their child and then after 1, 2.5–3, and 5 years. At year 8 and at 10–12-years, separate questionnaires were answered by both children and parents, and at 17–19 by the teenagers and at 23–25 years of age by the young adults. At every follow-up, biological samples were collected. Information was given to the parents before the birth of their child; see Ethics Approval and Consent to Participate below. When the children were 5-years old, they also received a short brochure describing the ABIS study. At 8-years of age, the children received further information through the ABIS website in a special section for children. The web pages contained more in-depth information about the study, and clarified that the study aimed to identify factors leading to type 1 diabetes. The children received their first own questionnaire when they were 8-years old.

The children received a more detailed letter of information prior to the 10–12-year data collection, and an information video was provided on the website. Initially, questionnaires were distributed to both children and their parents, and a hair sample was collected from the child. This initial data collection was completed with the assistance of schools, as research materials were distributed to them following the approval of the headmaster and class teachers. The parents were requested to receive a package containing written information (including the video link) and questionnaires from the children. Parents who did not wish their child to participate were requested to complete the form and submit it to the teacher. Therefore, children were enrolled in the 10–12-year follow-up after either (a) parental consent was implied by the parents’ completion of the parental questionnaire prior to the child’s participation in any ABIS activities at school or (b) a new parental written consent was obtained. A second data collection involved the distribution of mailed questionnaires to both children and parents in families that had completed at least two follow-up questionnaires prior to the 10–12-year-old child questionnaire and had collected at least one blood sample. More indepth information are available at the ABIS website (www.abis-studien.se).

Physical activity was classified as Total Activity Score (TAS) based on several variables including screen time (measured hourly, daily, and weekly), time spent reading books or journals, activity during child daycare, child in motion (such as jumping, running, outdoor play), exercise in school and leisure times, frequency of exercise leading to sweating, and average daily steps taken as an adult. Detailed descriptions of the questions and variables used to calculate the TAS are provided in the Electronic Supplementary Material (ESM).

A normalization technique was used to calculate the TAS [14, 15], with the objective of rescaling all physical activity-related variables to a common range between 0 and 1. To ensure that higher values reflected greater levels of physical activity, sedentary behavior variables such as screen time and time spent reading were reverse-coded prior to normalization. In contrast, variables directly related to physical activity (e.g., outdoor play, structured exercise, and daily step count) were normalized without reversal. Following these adjustments, all normalized values were averaged (summed and divided by the number of included variables) to produce the final TAS.

For questions related to hours per week, normalization was achieved by dividing by the maximum possible value (168 h, representing a week). Examples include questions like “How many hours per week is/was the child at the day care center?”. For questions regarding hours per day, values were normalized by dividing by 24 h. Examples include questions like “How many hours per day, on average, is the child active (playing, running around)?”. For frequency per week, values were normalized by dividing by 7 (the number of days in a week). Examples include questions like “How many times a week do you exercise for at least 30 min to start sweating?”. Total steps were normalized by dividing by a maximum value, such as 10,000 steps, for the question “How many steps have you taken in the last 24 h?”. Steps related questions were introduced in the later waves of data collection (at age 17–23) when mobile-based tracking tools had become commonly available. Participants were instructed to refer to their phone’s built-in pedometer and screen time logs to provide accurate responses.

Moreover, the following questions were used as variables in this study: sex of the child (male/female), child participation in sports, child lives with (both parents or single mother/father or other), accommodation type (living in houses, apartments, others), siblings, pets (dog) at home, parental smoking habits, parental education status, parental employment status (full time/part time), sleep quality of child according to the parents, and child health perception according to the parents.

### Statistics

**Fig. 1:**
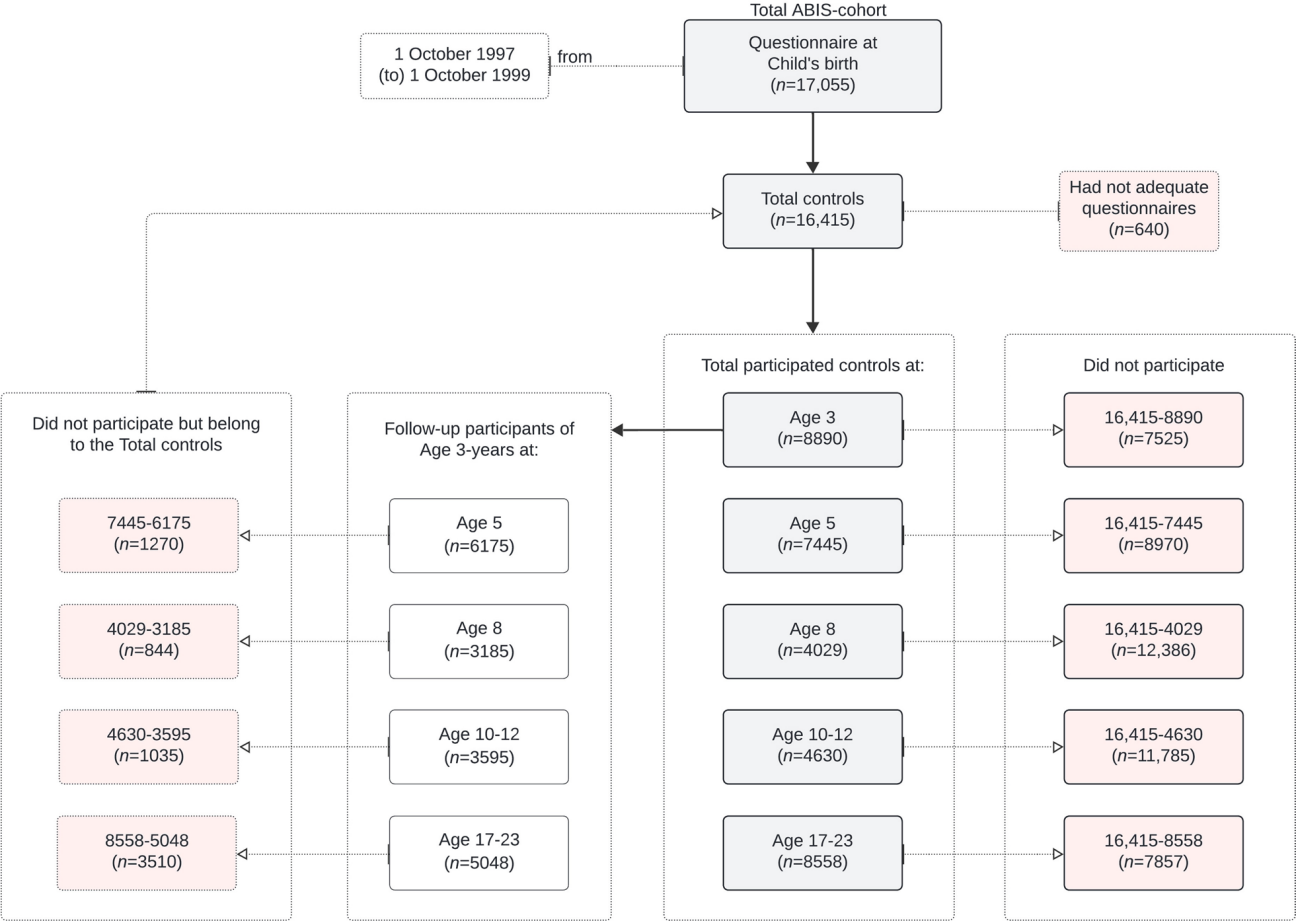
The All Babies in Southeast Sweden (ABIS) study sample

In this study, physical activity was examined at five specific age groups within the ABIS cohort: 3-years, 5-years, 8-years, 10–12 years, and 17–23 years (age group 17–19 and 23–25 was combined as one group called 17–23 years). The number of participants at each follow-up is detailed in Fig. 1, with 8890 participants at 3 years, 7445 at 5 years, 4029 at 8 years, 4630 at 10–12 years, and 8558 at 17–23 years. For each age group, the TAS variable was created by combining the normalized values of selected physical activity variables and dividing it by the total number of variables. To ensure the internal consistency of the TAS variable, Cronbach’s alpha (α) was computed at each age group based on the normalized variables. Participants were included if they had valid physical activity data at age 3 and at least one follow-up point (ages 5, 8, 10–12, or 17–23).

Children were classified into two physical activity groups, namely Less active group and High active group, by using a z-score-based classification method rather than arbitrary percentile cutoffs. Participants with TAS values less than 0.5 standard deviations (SD σ) below the group mean (TAS < μ − 0.5σ) were classified as Less active, and those with (TAS ≥ μ − 0.5σ) were classified as High active. This approach aligns with standard statistical practice, and avoids the subjectivity of arbitrary cutoffs, and ensures consistent classification across age groups. Under this threshold, approximately 31% of participants were categorized as Less active and 69% as High active within each age group. In addition, this approach supports and enhances the interpretability, comparability, and methodological precision of the classification scheme. More details are provided in the ESM including mean TAS, SD σ, Cronbach’s alpha (α), Shapiro–Wilk Stat, and classification thresholds.

The statistical analysis was conducted using IBM SPSS Statistics (v29.0) and Python (v3.12) with the Scikit-learn library (v1.4). Descriptive statistics were used to summarize participant characteristics and important variables. The chi-squared test was used to compare the variables between the cases and controls. Imputation was used to deal with missing values [15]. Mode imputation was applied for TAS variables within each age group. This involved replacing missing values with the most frequently occurring values within the same age group. The proportion of missing data per variable was low (< 5%). The multivariate approach likely utilised logistic regression analysis to examine the associations (odds ratio, 95% confidence interval) between the dependent variable (activity score) and independent variables. A *p* value below 0.05 was considered to be statistically significant.

### Ethics

The parents of the children participating in this study were given oral, written, and video information before giving their informed consent to participate in ABIS which was approved by the research ethics committees at Linköping University (Dnr 96-287, Dnr 99-321, and Dnr 03-092) and Lund University (LU 83-97) in Sweden. Connection of the ABIS registers to National Registers was approved by the Research Ethics Committee in Linköping (Dnr 2013/253-32). The children were given special information, both oral and written and website information at the age of 5 years and then before their questionnaires at the age of 8 and 11 years of age as described in the ‘Methods’ section.

### Data and Resource Availability

The data that support the findings of this study are available from the corresponding author, but ethical restrictions apply to the availability of these data, which therefore are not publicly available.

## Results

**Fig. 2:**
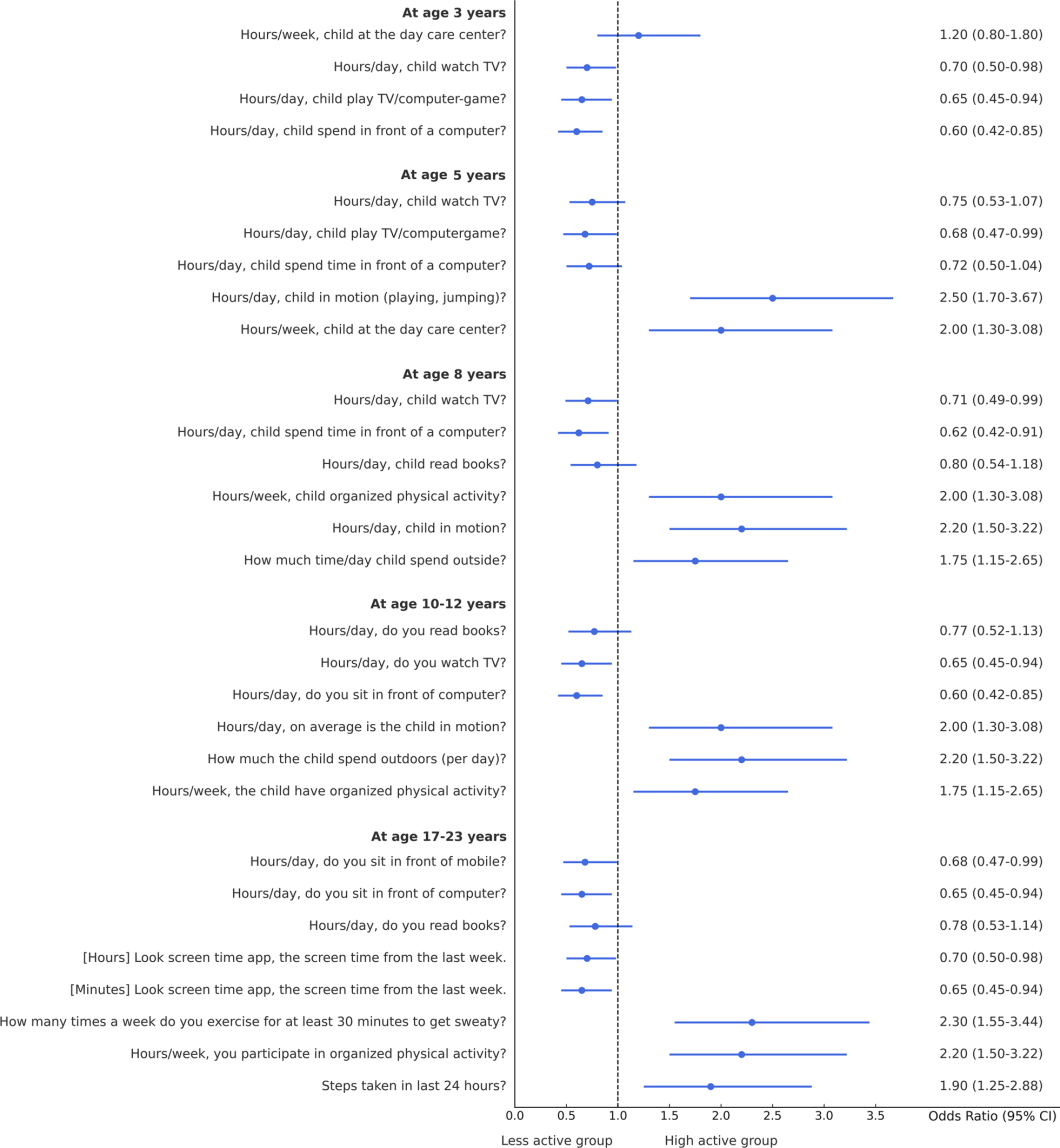
Odds ratio analysis of questions/variables associated with Total Activity Score (TAS) in each age group

In the total ABIS population, a total of 8890 individuals (54.2%) took part in the study at the age of 3. Among them, 51% were males and 49% were females. Table 1 shows the descriptive analysis of all variables used in this study and distinct patterns across ages, with males comprising slightly higher populations at younger ages, but with a shift towards females in the 17–23 age groups likely (46% males and 54% females). Figure 2 shows the classification results with odds ratios (OR) and confidence interval (CI) for each physical activity variable in each age group (by considering TAS as a dependent variable) to show the high and low physical activity in each physical activity variable. The results of various factors associated with levels of physical activity among children and adolescents were classified into Less active group (Q1) and High active group (Q2) (Tables 2, 3, 4, 5, 6) by considering TAS as a dependent variable. TAS was calculated separately for Q1 and Q2 within each age group (see ESM) to identify factors associated with activity levels at each time point. Subsequently, follow-up analyses were conducted (Tables 2, 3, 4, 5, 6) at age 5 with 6175 participants, 3185 at age 8, 3595 at age 10–12, and 5048 at age 17–23.

**Table 1:** Descriptive analysis of the variables in each age group

Variables	Age 3 (*n* = 8890) (%)	Age 5 (*n* = 7445) (%)	Age 8 (*n* = 4029) (%)	Age 10–12 (*n* = 4630) (%)	Age 17–23 (*n* = 8558) (%)
Sex					
Male	4561 (51)	3850 (52)	2100 (52)	2391 (51)	3956 (46)
Female	4329 (49)	3595 (48)	1929 (48)	2239 (49)	4602 (54)
Total activity score					
Q1	2756 (31)	2308 (31)	1249 (31)	1435 (31)	2653 (31)
Q2	6134 (69)	5137 (69)	2780 (69)	3195 (69)	5905 (69)
Sports participation					
Yes	–	–	3470 (86)	4058 (88)	5505 (64)
No	–	–	559 (14)	572 (12)	3053 (36)
Child lives with					
Both parents	7881 (88)	6391 (86)	3164 (79)	3716 (80)	5014 (59)
Split custody	297 (3)	218 (3)	210 (5)	241 (6)	566 (6)
Mother (single)	191 (2)	195 (2)	92 (2)	150 (3)	171 (2)
Father (single)	115 (1)	297 (4)	343 (9)	153 (3)	316 (4)
Other	406 (5)	344 (5)	220 (5)	370 (8)	2491 (29)
Siblings					
0	3531 (40)	1802 (24)	304 (8)	457 (10)	–
1	3192 (36)	1200 (16)	2091 (52)	1781 (38)	–
2	1340 (15)	3983 (54)	1267 (31)	1799 (39)	–
≥3	827 (9)	460 (6)	367 (9)	593 (13)	–
Accommodation type					
House	6866 (77)	2070 (28)	3420 (85)	3800 (82)	–
Apartment	1463 (17)	2784 (37)	370 (9)	274 (6)	–
Other	561 (6)	2591 (35)	239 (6)	556 (12)	–
Animal pet (dog)					
Yes	1739 (20)	5914 (79)	1405 (35)	1859 (40)	–
No	7151 (80)	1531 (21)	2624 (65)	2771 (60)	–
Mother (smoking)					
Yes	1217 (14)	896 (12)	623 (15)	855 (18)	1486 (17)
No	7673 (86)	6549 (88)	3406 (85)	3775 (82)	7072 (83)
Father (smoking)					
Yes	1102 (12)	929 (12)	542 (13)	928 (20)	507 (6)
No	7788 (88)	6516 (88)	3487 (87)	3702 (80)	8051 (94)
Mother (education level)					
≤9	1032 (12)	723 (10)	210 (5)	456 (10)	1123 (13)
>9 and ≤12	4824 (54)	4125 (55)	1912 (48)	1449 (31)	5366 (63)
>12	3034 (34)	2597 (35)	1907 (47)	2725 (59)	2069 (24)
Father (education level)					
≤9	1061 (12)	916 (12)	504 (13)	756 (16)	1947 (23)
>9 and ≤12	5070 (57)	4447 (60)	2391 (59)	1627 (35)	2042 (24)
>12	2759 (31)	2082 (28)	1134 (28)	2247 (49)	4569 (53)
Mother (working status)					
Full time	876 (10)	739 (10)	421 (11)	3109 (67)	6945 (81)
Part time (0–50%)	2832 (32)	1967 (26)	2593 (64)	483 (11)	946 (11)
Part time (51–90%)	5182 (58)	4739 (64)	1015 (25)	1038 (22)	667 (8)
Father (working status)					
Full time	8281 (93)	6671 (90)	1185 (29)	3727 (81)	7990 (93)
Part time (0–50%)	206 (2)	315 (4)	826 (21)	245 (5)	277 (3)
Part time (51–90%)	403 (5)	459 (6)	2018 (50)	658 (14)	291 (4)
Child sleep quality					
Good	7180 (81)	6065 (81)	3724 (92)	4076 (88)	5913 (69)
Bad	1710 (19)	1380 (19)	305 (8)	554 (12)	2645 (31)
Child health perception					
Good	8002 (90)	6103 (82)	3815 (95)	4416 (95)	6031 (70)
Bad	888 (10)	1342 (18)	214 (5)	214 (5)	2527 (30)

**Table 2:**
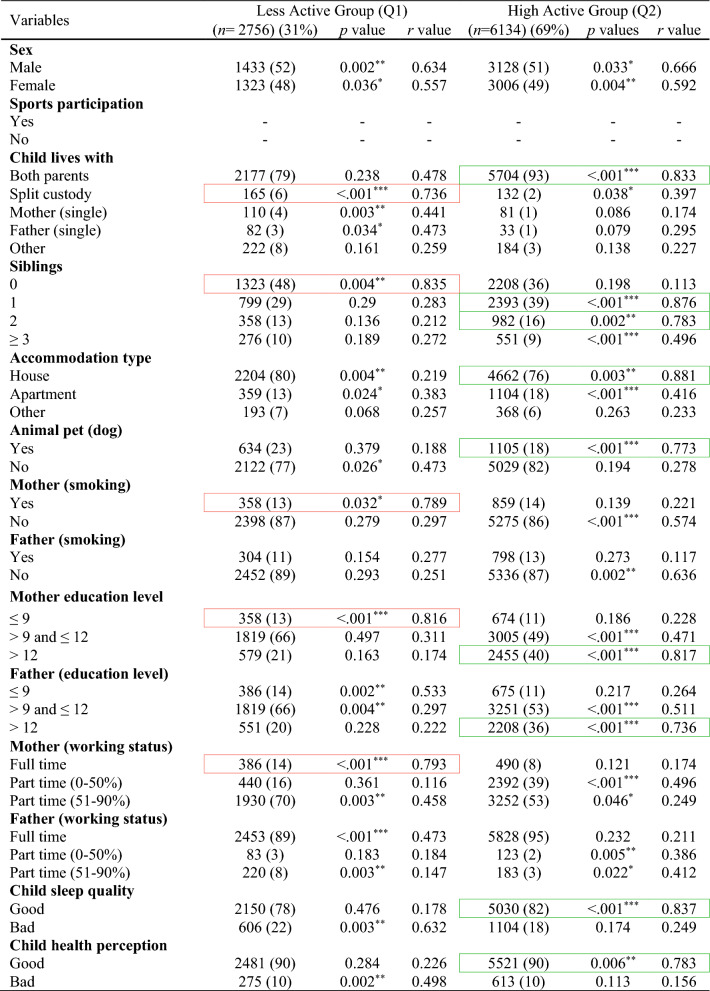
Descriptive analysis of age 3-years participants (*n* = 8890)

Red box, strong correlation in Q1; green box, strong correlation in Q2

**Table 3:**
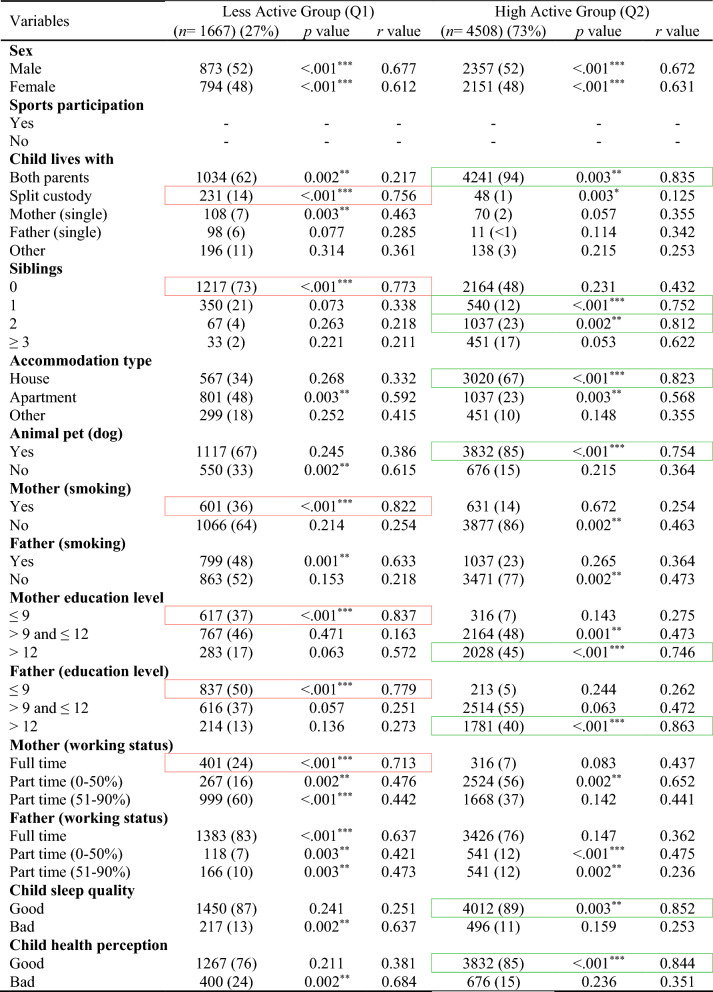
Follow-up analysis of age 3-year participants at age 5-years (*n* = 6175)

Red box, strong correlation in Q1; green box, strong correlation in Q2

**Table 4:**
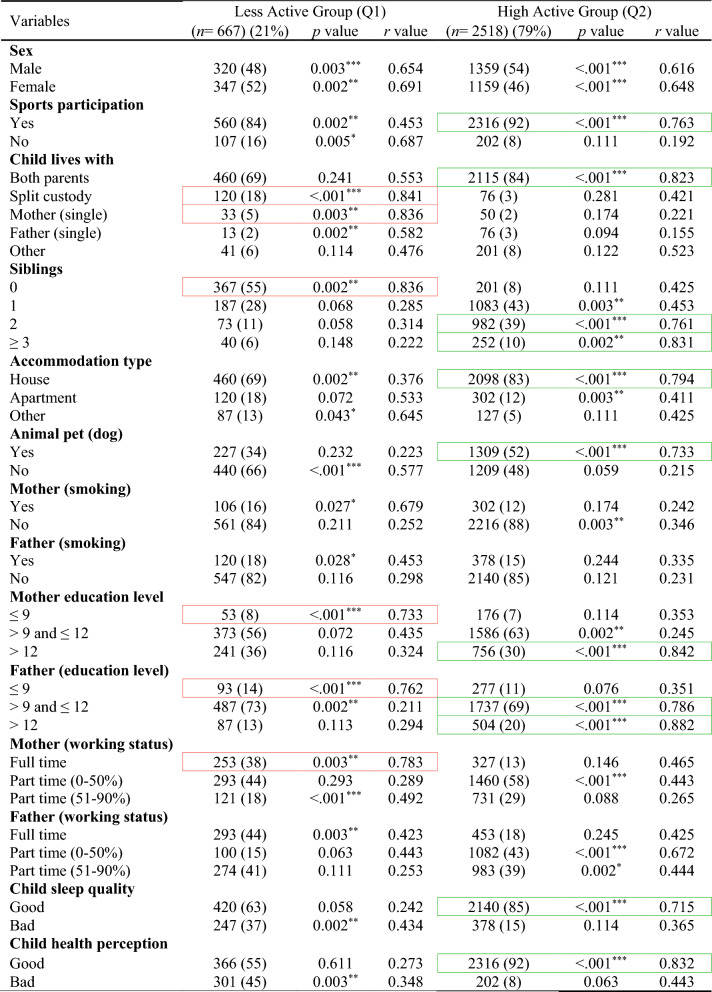
Follow-up analysis of age 3-year participants at age 8-years (*n* = 3185)

Red box, strong correlation in Q1; green box, strong correlation in Q2

**Table 5:**
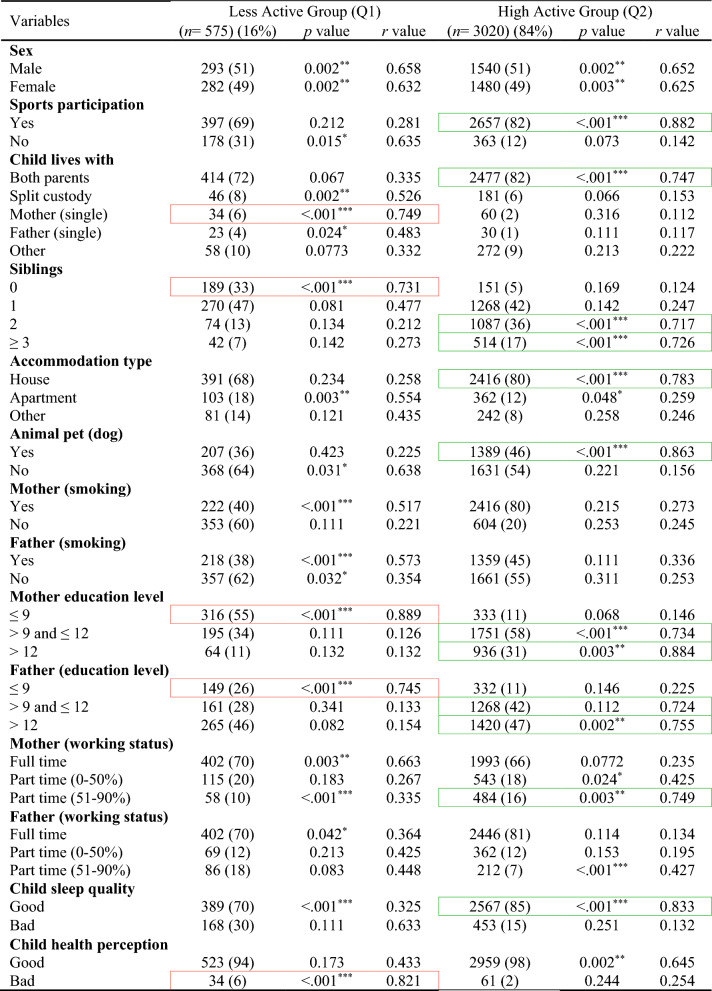
Follow-up analysis of age 3-year participants at age 10–12-years (*n* = 3595)

Red box, strong correlation in Q1; green box, strong correlation in Q2

**Table 6:**
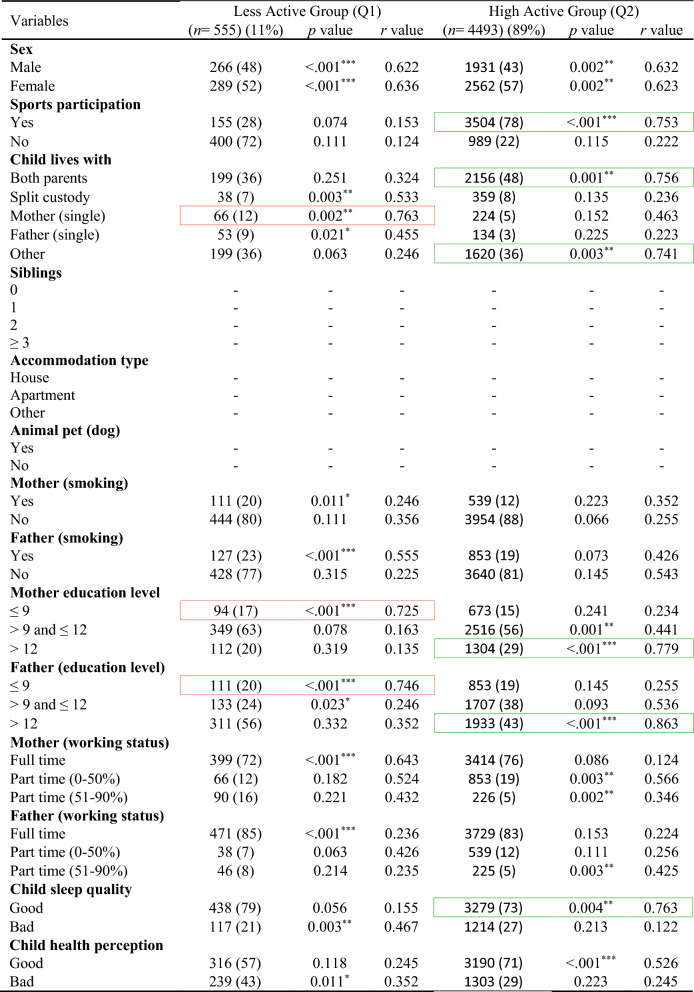
Follow-up analysis of age 3-year participants at age 17–23-years (*n* = 5048)

Red box, strong correlation in Q1; green box, strong correlation in Q2

The results at age 3-years (Table 2) shows that children classified as highly active were associated with factors like living with both parents (*r* = 0.833, *p* < 0.001), ≥1 siblings (*r* = 0.876, *p* < 0.001), accommodation type houses (*r* = 0.881, *p* = 0.003), dog as a pet (*r* = 0.773, *p* < 0.001), highly educated parents (*r* = 0.817, *p* < 0.001), good sleep quality (*r* = 0.837, *p* < 0.001), and good health perceptions (*r* = 0.786, *p* = 0.006). The factors lead to less active children are split custody (*r* = 0.736, *p* < 0.001), no siblings (*r* = 0.835, *p* = 0.004), mother smoking (*r* = 0.789, *p* = 0.032), less educated mothers (*r* = 0.816, *p* < 0.001), and mother working as full-time (*r* = 0.793, *p* < 0.001).

A similar pattern of associated factors was observed at the follow-up age 5-years (Table 3). Children were highly active with factors involves: living with both parents (*r* = 0.835, *p* = 0.003), ≥1 siblings (*r* = 0.812, *p* = 0.002), accommodation type houses (*r* = 0.823, *p* < 0.001), dog as a pet (*r* = 0.754, *p* < 0.001), highly educated parents (*r* = 0.746, *p* < 0.001), good sleep quality (*r* = 0.852, *p* = 0.003), and good health perceptions (*r* = 0.844, *p* < 0.001). Factors with less active behaviour involves: split custody (*r* = 0.756, *p* < 0.001), no siblings (*r* = 0.773, *p* < 0.001), mother smoking (*r* = 0.822, *p* < 0.001), less educated parents (*r* = 0.837, *p* < 0.001), and full-time working mother (*r* = 0.713, *p* < 0.001).

The pattern of associated factors observed at age 5-years persisted at the follow-up assessment at age 8-years (Table 4) but sport participation became an important factor associated to high physical activity. High active group factor involves: sport participation (*r* = 0.763, *p* < 0.001), living with both parents (*r* = 0.823, *p* < 0.001), ≥2 siblings (*r* = 0.832, *p* = 0.002), accommodation type houses (*r* = 0.794, *p* < 0.001), dog as a pet (*r* = 0.733, *p* < 0.001), highly educated parents (*r* = 0.842, *p* < 0.001), good sleep quality (*r* = 0.715, *p* < 0.001), and good health perceptions (*r* = 0.832, *p* < 0.001). Less active group factor involves: split custody (*r* = 0.841, *p* < 0.001), single mother (*r* = 0.836, *p* = 0.003), no siblings (*r* = 0.836, *p* = 0.002), less educated parents (*r* = 0.733, *p* < 0.001), and full-time working mother (*r* = 0.783, *p* = 0.003).

The factors linked to higher physical activity levels at age 8-years continued to show similar associations at age 10–12-years (Table 5). Children were highly active with factors involves: sport participation (*r* = 0.882, *p* < 0.001), living with both parents (*r* = 0.747, *p* < 0.001), ≥2 siblings (*r* = 0.717, *p* < 0.001), accommodation type houses (*r* = 0.783, *p* < 0.001), dog as a pet (*r* = 0.863, *p* < 0.001), highly educated parents (*r* = 0.734, *p* < 0.001), and good sleep quality (*r* = 0.833, *p* < 0.001). Less active group factor involves: single mother (*r* = 0.749, *p* < 0.001), no siblings (*r* = 0.731, *p* < 0.001), less educated parents (*r* = 0.889, *p* < 0.001), and bad health perception of child (*r* = 0.821, *p* < 0.001). At age 17–23-years (Table 6), some factors showed notable changes compared to earlier time points, such as high active group includes sport participation (*r* = 0.753, *p* < 0.001), living with both parents (*r* = 0.756, *p* = 0.001), living with others (*r* = 0.741, *p* = 0.003), highly educated parents (*r* = 0.779, *p* < 0.001), and good sleep quality (*r* = 0.763, *p* = 0.004). Less active group includes: single mother (*r* = 0.763, *p* = 0.002), less educated mother (*r* = 0.725, *p* < 0.001), and less educated father (*r* = 0.746, *p* < 0.001).

## Discussion

In our longitudinal analysis of the Q2 group from age 3 to 23 years, we found that individuals who were highly active in early adulthood (ages 17–23) had also been active during childhood. Several key factors were strongly associated with higher physical activity levels in children, including living with both parents in a house/villa and having a pet dog. Additionally, parental factors such as higher education and part-time employment (0–50%) were significantly correlated with children’s and adolescents’ elevated physical activity levels. Another major influence on sustained high activity into adulthood was consistent parental reporting of good sleep quality and positive health perception throughout childhood.

Conversely, our investigation of the Q1 group representing children with lower physical activity revealed associations with living in split custody arrangements, lower parental education, and part-time employment (51–90%). Maternal smoking also emerged as a significant factor, strongly correlated with sedentary behavior. These findings suggest that lower educational attainment and potential job-related stress (i.e., challenges in maintaining work-life balance) may negatively impact a child’s physical activity behavior. Encouragingly, as parental assessments of a child’s health perception and sleep quality improved, sedentary behavior appeared to decline.

A notable increase in sports participation was observed after age 8. This period, known as middle childhood, is a critical developmental stage for acquiring physical, social, and cognitive skills conducive to team sports participation [16, 17]. Our results, along with previous research, underscore the importance of structured sports programs in promoting physical activity among children aged 8 and older.

Regarding family structure, our findings align with prior studies [18–20] that report a decrease in two-parent households as children age. Notably, living with both parents is consistently associated with higher levels of physical activity in children [21–23]. Similarly, higher parental education was strongly linked to increased physical activity, as more educated parents may be more likely to value and support active lifestyles in their children [24, 25]. Children from families with lower educational attainment may benefit from targeted encouragement to participate in sports, potentially supported by financial assistance programs.

Our study also found a decline in both maternal and paternal smoking rates across age groups, an encouraging trend likely reflecting broader public health efforts. Recent research [26, 27] has documented similar declines, attributing them to increased health awareness and the promotion of healthy behaviors, including physical activity in children. Lower parental smoking is generally associated with healthier household environments and more physically active children [28, 29].

Across all age groups, we observed a common employment pattern: fathers were typically employed full-time, while mothers worked part-time (0–50%). This distribution may play a role in shaping children’s physical activity by modeling work-life balance and enabling access to sports and recreational opportunities. Uthede et al. [30] highlights that while full-time employment can limit direct parental involvement in children’s activities, it may also provide the financial means to support structured physical opportunities.

Finally, consistent with our findings, previous studies [31–34] emphasize the crucial role of adequate sleep and positive health perception in promoting physical activity. Sleep quality, in particular, has been linked to both physical and cognitive development, which can in turn influence children’s energy levels and willingness to participate in physical activities. Our results support the notion that high levels of physical activity in early life tend to track into adulthood, reinforcing the importance of early interventions to promote lifelong health behaviors.

Moreover, our longitudinal findings align closely with previous Nordic research demonstrating the persistence of physical activity from childhood into adulthood. In the Cardiovascular Risk in Young Finns Study, Telama et al. [35] reported moderate to high tracking coefficients for physical activity from preschool age into early adulthood, especially among males, consistent with our observation of stable high activity in the Q2 group from ages 3 to 23. Similarly, Rovio et al. [36] identified five distinct physical activity trajectories from childhood through midlife, with the “persistently active” trajectory linked to higher academic achievement and education, paralleling our findings regarding the influence of parental education and early-life factors. Further, Yang et al. [37] showed that parental physical activity trajectories strongly predict offsprings activity patterns into adulthood, supporting our evidence that parental behaviors, including non-smoking, employment stability, and healthy sleep routines, significantly correlate with children’s long-term activity levels.

### Strength and Limitations

This research has significant strengths, primarily due to its foundation on a comprehensive birth cohort study that encompasses a broad segment of the general population with a high response rate. By utilizing a large database, the study ensures that the findings are both robust and widely applicable, enhancing the reliability and generalizability of the results. However, this study has limitations, as it includes responses only from motivated individuals particularly interested in participating in the ABIS project. This may mean that the overall degree of physical activity is higher than in a general population, but this should not influence the investigation of which factors were associated with the degree of physical activity. Furthermore, some variables related to children’s physical activity levels are missing in the adult age group which poses a challenge for accurately assessing the physical activity patterns in adults.

## Conclusion

In conclusion, our study identifies several key factors associated with physical activity levels among children and adolescents, offering valuable insights for targeted public health interventions. Children from families with fewer resources such as those in split households or with lower parental education may require additional support to participate in organized sports.

## Supplementary Information

Supplementary Material 1.

## Data Availability

Data can be obtained from the corresponding author on reasonable request after ethical approval.
