# Supplementary material for: Early Life Physical Activity Patterns and Its Survival to Adult Activity Levels: The Longitudinal ABIS Study
Source: Sports Med Open. 2025 Nov 21;11:138. doi: 10.1186/s40798-025-00934-6 (PMC12638552; doi:10.1186/s40798-025-00934-6)
Supplement: Supplementary file 1 — Supplementary Material 1. [file 40798_2025_934_MOESM1_ESM.pdf]

1. Questionnaire:

Table (1a): The questionnaire used to measure the Total Activity Score (TAS).

At age 3 years
Hours/week, child at the day care center?
Hours/day, child watch TV?
Hours/day, child play TV/computer -games?
Hours/day, child spend in front of a computer?
At age 5 years
Hours/day, child watch TV?
Hours/day, child play TV/computergame?
Hours/day, child spend time in front of a computer?
Hours/day, child in motion (playing, jumping)?
Hours/week, child at the day care center?
At age 8 years
Hours/day, child watch TV?
Hours/day, child spend time in front of a computer?
Hours/day, child read books?
Hours/week, child organized physical activity?
Hours/day, child in motion?
How much time/day child spend outside?
At age 10-12 years
Hours/day, do you read books?
Hours/day, do you watch TV?
Hours/day, do you sit in front of computer?
Hours/day, on average is the child in motion?
How much the child spends outdoors (per day)?
Hours/week, the child has organized physical activity?
At age 17-23 years
Hours/day, do you sit in front of mobile?
Hours/day, do you sit in front of computer?
Hours/day, do you read books?
[Hours] Look screen time app, the screen time from the last week.
[Minutes] Look screen time app, the screen time from the last week.
How many times a week do you exercise for at least 30 minutes so you get sweaty?
Hours/week, you participate in organized physical activity?
Steps taken in last 24 hours?

2. Descriptive and Sensitivity analysis of TAS

We evaluated the internal consistency of TAS by simulating physical activity variables and calculating Cronbach's Alpha (α). All age groups showed excellent internal consistency (α > 0.98), validating the use of TAS as a reliable composite measure of physical activity.

Table (2a): Descriptive characteristics and Sensitivity analysis at each age point.

Age	N (Participants)	Mean TAS	SD (σ)	Shapiro-Wilk Stat	Cronbach's Alpha
Age 3	8890	49.98	10.09	0.99	0.98
Age 5	7445	52.06	8.96	0.99	0.98
Age 8	4029	54.12	8.02	0.99	0.98
Age 10–12	4630	55.89	6.85	0.99	0.98
Age 17–23	8558	57.99	6.04	0.99	0.98

We selected the threshold of (μ − 0.5σ) for classifying children as "Less Active" based on its grounding in standard deviation theory under the assumption of normality. In a standard normal distribution, approximately 31% of values

fall below this threshold, making it a statistically interpretable and non-arbitrary point of division. This avoids the subjectivity associated with percentile-based cutoffs and ensures comparability across age groups. Further, applying stricter thresholds ($\mu - 0.75\sigma$ or $\mu - 1\sigma$) led to a lower proportion of participants being classified as "Less Active" and the relative proportions also not stable across age groups. The use of ($\mu - 0.5\sigma$), resulting in a consistent ~31% classification rate, therefore provides both theoretical justification, empirical stability, and strengthening the methodology of the analysis.

Table (2b): Threshold comparison at each age point.

Age	Less Active Group Percentage		
	$\mu - 0.5\sigma$	$\mu - 0.75\sigma$	$\mu - 1\sigma$
Age 3	31.00	22.93	11.87
Age 5	31.00	17.27	11.86
Age 8	31.00	16.15	9.73
Age 10–12	30.99	20.94	13.91
Age 17–23	31.00	21.11	15.82

3. Descriptive analysis of TAS in each age group

Table (3a): At age 3–years (*N*= 8990). *(Red box) strong corelation in Q1; (Green box) strong corelation in Q2.*

Variables	Less Active Group (Q1)			Highly Active Group (Q2)		
	(*n*= 2756) (%)	*p* value	*r* value	(*n*=6134) (%)	*p* values	*r* value
**Sex**						
Male	1433 (52)	0.002**	0.634	3128 (51)	0.033*	0.666
Female	1323 (48)	0.036*	0.557	3006 (49)	0.004**	0.592
**Sports participation**						
Yes	-	-	-	-	-	-
No	-	-	-	-	-	-
**Child lives with**						
Both parents	2177 (79)	0.238	0.478	5704 (93)	<.001***	0.833
Split custody	165 (6)	<.001***	0.736	132 (2)	0.038*	0.397
Mother (single)	110 (4)	0.003**	0.441	81 (1)	0.086	0.174
Father (single)	82 (3)	0.034*	0.473	33 (1)	0.079	0.295
Other	222 (8)	0.161	0.259	184 (3)	0.138	0.227
**Siblings**						
0	1323 (48)	0.004**	0.835	2208 (36)	0.198	0.113
1	799 (29)	0.29	0.283	2393 (39)	<.001***	0.876
2	358 (13)	0.136	0.212	982 (16)	0.002**	0.783
≥ 3	276 (10)	0.189	0.272	551 (9)	<.001***	0.496
**Accommodation type**						
House	2204 (80)	0.004**	0.219	4662 (76)	0.003**	0.881
Apartment	359 (13)	0.024*	0.383	1104 (18)	<.001***	0.416
Other	193 (7)	0.068	0.257	368 (6)	0.263	0.233
**Animal pet (dog)**						
Yes	634 (23)	0.379	0.188	1105 (18)	<.001***	0.773
No	2122 (77)	0.026*	0.473	5029 (82)	0.194	0.278
**Mother (smoking)**						
Yes	358 (13)	0.032*	0.789	859 (14)	0.139	0.221
No	2398 (87)	0.279	0.297	5275 (86)	<.001***	0.574
**Father (smoking)**						
Yes	304 (11)	0.154	0.277	798 (13)	0.273	0.117
No	2452 (89)	0.293	0.251	5336 (87)	0.002**	0.636
**Mother education level**						
≤ 9	358 (13)	<.001***	0.816	674 (11)	0.186	0.228

> 9 and ≤ 12	1819 (66)	0.497	0.311	3005 (49)	<.001***	0.471
> 12	579 (21)	0.163	0.174	2455 (40)	<.001***	0.817
**Father (education level)**						
≤ 9	386 (14)	0.002**	0.533	675 (11)	0.217	0.264
> 9 and ≤ 12	1819 (66)	0.004**	0.297	3251 (53)	<.001***	0.511
> 12	551 (20)	0.228	0.222	2208 (36)	<.001***	0.736
**Mother (working status)**						
Full time	386 (14)	<.001***	0.793	490 (8)	0.121	0.174
Part time (0-50%)	440 (16)	0.361	0.116	2392 (39)	<.001***	0.496
Part time (51-90%)	1930 (70)	0.003**	0.458	3252 (53)	0.046*	0.249
**Father (working status)**						
Full time	2453 (89)	<.001***	0.473	5828 (95)	0.232	0.211
Part time (0-50%)	83 (3)	0.183	0.184	123 (2)	0.005**	0.386
Part time (51-90%)	220 (8)	0.003**	0.147	183 (3)	0.022*	0.412
**Child sleep quality**						
Good	2150 (78)	0.476	0.178	5030 (82)	<.001***	0.837
Bad	606 (22)	0.003**	0.632	1104 (18)	0.174	0.249
**Child health perception**						
Good	2481 (90)	0.284	0.226	5521 (90)	0.006**	0.783
Bad	275 (10)	0.002**	0.498	613 (10)	0.113	0.156

Table (3b): At age 5–years (*N*= 7445). *(Red box) strong corelation in Q1; (Green box) strong corelation in Q2.*

Variables	Less Active Group (Q1) (*n*= 2308) (%)	*p* value	*r* value	Highly Active Group (Q2) (*n*= 5137) (%)	*p* value	*r* value
**Sex**						
Male	1178 (51)	<.001***	0.736	2672 (52)	<.001***	0.637
Female	1130 (49)	<.001***	0.695	2465 (48)	<.001***	0.771
**Sports participation**						
Yes	-	-	-	-	-	-
No	-	-	-	-	-	-
**Child lives with**						
Both parents	1870 (81)	0.002**	0.268	4521 (88)	<.001***	0.873
Split custody	115 (5)	<.001***	0.875	103 (2)	0.213	0.278
Mother (single)	93 (4)	0.003**	0.542	102 (2)	0.153	0.227
Father (single)	91 (4)	0.248	0.174	206 (4)	0.215	0.192
Other	139 (6)	0.173	0.212	205 (4)	0.237	0.118
**Siblings**						
0	877 (38)	<.001***	0.881	925 (18)	0.313	0.215
1	277 (12)	0.219	0.284	923 (18)	<.001***	0.501
2	900 (39)	0.101	0.175	3083 (60)	0.003**	0.896
≥ 3	254 (11)	0.217	0.119	206 (4)	0.002**	0.683
**Accommodation type**						
House	323 (14)	0.116	0.137	1747 (34)	<.001***	0.885
Apartment	370 (16)	0.002**	0.534	2414 (47)	0.002**	0.677
Other	1615 (70)	0.172	0.281	976 (19)	0.271	0.144
**Animal pet (dog)**						
Yes	1754 (76)	0.185	0.172	4160 (81)	<.001***	0.773
No	554 (24)	0.003**	0.662	977 (19)	0.399	0.192
**Mother (smoking)**						
Yes	485 (21)	0.013*	0.703	411 (8)	0.273	0.111
No	1823 (79)	0.194	0.119	4726 (92)	0.003**	0.536
**Father (smoking)**						
Yes	415 (18)	0.011*	0.757	514 (10)	0.191	0.212
No	1893 (82)	0.113	0.184	4623 (89)	0.043*	0.573

**Mother education level**						
≤ 9	415 (18)	<.001***	0.818	308 (6)	0.371	0.113
> 9 and ≤ 12	1454 (63)	0.319	0.281	2671 (52)	0.002**	0.555
> 12	439 (19)	0.118	0.176	2158 (42)	<.001***	0.839
**Father (education level)**						
≤ 9	300 (13)	0.002**	0.727	616 (12)	0.154	0.174
> 9 and ≤ 12	1569 (68)	0.317	0.115	2878 (56)	0.137	0.135
> 12	439 (19)	0.159	0.138	1643 (32)	<.001***	0.885
**Mother (working status)**						
Full time	277 (12)	0.128	0.117	462 (9)	0.313	0.224
Part time (0-50%)	323 (14)	0.012*	0.163	1644 (32)	<.001***	0.718
Part time (51-90%)	1708 (74)	<.001***	0.575	3031 (59)	0.117	0.291
**Father (working status)**						
Full time	2100 (91)	<.001***	0.497	4571 (89)	0.288	0.116
Part time (0-50%)	161 (7)	0.003**	0.114	154 (3)	<.001***	0.554
Part time (51-90%)	47 (2)	0.238	0.247	412 (8)	0.039*	0.279
**Child sleep quality**						
Good	1801 (78)	0.179	0.188	4264 (83)	0.002**	0.846
Bad	507 (22)	<.001***	0.428	873 (17)	0.216	0.173
**Child health perception**						
Good	1685 (73)	0.277	0.231	4418 (86)	<.001***	0.856
Bad	623 (27)	0.021*	0.491	719 (14)	0.172	0.247

Table (3c): At age 8–years (*N*= 4029). *(Red box) strong corelation in Q1; (Green box) strong corelation in Q2.*

Variables	Less Active Group (Q1)			Highly Active Group (Q2)		
	(*n*= 1249) (%)	*p* value	*r* value	(*n*= 2780) (%)	*p* value	*r* value
**Sex**						
Male	599 (48)	<.001***	0.634	1501 (54)	<.001***	0.872
Female	650 (52)	0.002**	0.773	1279 (46)	<.001***	0.758
**Sports participation**						
Yes	1024 (82)	0.007**	0.167	2446 (88)	<.001***	0.887
No	225 (18)	0.005*	0.433	334 (12)	0.113	0.172
**Child lives with**						
Both parents	912 (73)	0.141	0.181	2252 (81)	<.001***	0.839
Split custody	99 (8)	<.001***	0.862	111 (4)	0.136	0.293
Mother (single)	37 (3)	0.002**	0.769	55 (2)	0.273	0.118
Father (single)	37 (3)	0.004**	0.398	306 (11)	0.396	0.174
Other	164 (13)	0.172	0.237	56 (2)	0.153	0.266
**Siblings**						
0	137 (11)	0.003**	0.759	167 (6)	0.188	0.217
1	674 (54)	0.101	0.191	1417 (51)	0.024*	0.496
2	350 (28)	0.008**	0.116	917 (33)	<.001***	0.763
≥ 3	88 (7)	0.391	0.274	279 (10)	0.013*	0.891
**Accommodation type**						
House	974 (78)	0.008**	0.221	2446 (88)	0.002**	0.875
Apartment	175 (14)	0.176	0.164	195 (7)	0.002**	0.447
Other	100 (8)	0.012*	0.431	139 (5)	0.185	0.195
**Animal pet (dog)**						
Yes	349 (28)	0.27	0.263	1056 (38)	0.002**	0.778
No	900 (72)	<.001***	0.347	1724 (62)	0.192	0.111
**Mother (smoking)**						
Yes	262 (2)	0.036*	0.775	361 (13)	0.398	0.291
No	987 (79)	0.111	0.113	2419 (87)	0.018*	0.227
**Father (smoking)**						
Yes	237 (19)	0.036*	0.437	305 (11)	0.182	0.223

No	1012 (81)	0.312	0.172	2475 (89)	0.219	0.154
**Mother education level**						
≤ 9	99 (8)	<.001***	0.794	111 (4)	0.394	0.153
> 9 and ≤ 12	688 (55)	0.273	0.233	1224 (44)	0.017*	0.127
> 12	462 (37)	0.136	0.281	1445 (52)	<.001***	0.882
**Father (education level)**						
≤ 9	199 (16)	<.001***	0.771	305 (11)	0.129	0.116
> 9 and ≤ 12	612 (49)	0.027*	0.284	1779 (64)	0.194	0.146
> 12	438 (35)	0.101	0.113	696 (25)	<.001***	0.835
**Mother (working status)**						
Full time	199 (16)	0.002**	0.695	222 (8)	0.273	0.216
Part time (0-50%)	786 (63)	0.165	0.116	1807 (65)	<.001***	0.473
Part time (51-90%)	264 (21)	<.001***	0.498	751 (27)	0.182	0.118
**Father (working status)**						
Full time	324 (26)	0.034*	0.494	861 (31)	0.271	0.117
Part time (0-50%)	187 (15)	0.274	0.139	639 (23)	<.001***	0.731
Part time (51-90%)	738 (59)	0.171	0.225	1280 (46)	0.133	0.213
**Child sleep quality**						
Good	1111 (89)	0.256	0.153	2613 (94)	0.003**	0.781
Bad	138 (11)	0.027*	0.447	167 (6)	0.149	0.116
**Child health perception**						
Good	1174 (94)	0.139	0.118	2641 (95)	0.002**	0.857
Bad	75 (6)	0.002**	0.397	139 (5)	0.271	0.213

Table (3d): At age 10–12–years (*N*= 4630). *(Red box) strong corelation in Q1; (Green box) strong corelation in Q2.*

Variables	Less Active Group (Q1)			Highly Active Group (Q2)		
	(*n*= 1435) (%)	*p* value	*r* value	(*n*= 3195) (%)	*p* value	*r* value
**Sex**						
Male	761 (53)	0.003**	0.735	1630 (51)	<.001***	0.663
Female	674 (47)	<.001***	0.697	1565 (49)	0.002**	0.638
**Sports participation**						
Yes	1119 (78)	0.332	0.281	2939 (92)	<.001***	0.885
No	316 (22)	0.029*	0.301	256 (8)	0.276	0.193
**Child lives with**						
Both parents	1033 (72)	0.271	0.233	2683 (84)	<.001***	0.798
Split custody	114 (8)	<.001***	0.572	127 (4)	0.057	0.183
Mother (single)	86 (6)	0.002**	0.716	64 (2)	0.237	0.111
Father (single)	57 (4)	0.037*	491	96 (3)	0.183	0.163
Other	145 (10)	0.073	0.117	225 (7)	0.115	0.201
**Siblings**						
0	330 (23)	<.001***	0.701	127 (4)	0.106	0.112
1	631 (44)	0.083	0.103	1150 (36)	0.298	0.174
2	360 (25)	0.317	0.111	1439 (45)	<.001***	0.763
≥ 3	114 (8)	0.263	0.273	479 (15)	<.001***	0.771
**Accommodation type**						
House	1148 (80)	0.371	0.237	2652 (83)	<.001***	0.679
Apartment	114 (8)	0.002**	0.552	160 (5)	0.050*	0.217
Other	173 (12)	0.283	0.173	383 (12)	0.163	0.113
**Animal pet (dog)**						
Yes	517 (36)	0.213	0.254	1342 (42)	<.001***	0.838
No	918 (64)	0.036*	0.747	1853 (58)	0.211	0.122
**Mother (smoking)**						
Yes	344 (24)	<.001***	0.511	511 (16)	0.319	0.174
No	1091 (76)	0.113	0.193	2684 (84)	0.582	0.201

**Father (smoking)**						
Yes	258 (18)	<.001***	0.281	670 (21)	0.237	0.174
No	1177 (82)	0.016*	0.114	2525 (79)	0.311	0.118
**Mother education level**						
≤ 9	201 (14)	<.001***	0.889	255 (8)	0.398	0.136
> 9 and ≤ 12	459 (32)	0.127	0.174	990 (31)	<.001***	0.764
> 12	775 (54)	0.363	0.157	1950 (61)	0.002**	0.564
**Father (education level)**						
≤ 9	373 (26)	<.001***	0.773	383 (12)	0.371	0.116
> 9 and ≤ 12	445 (31)	0.254	0.164	1182 (37)	0.184	0.154
> 12	617 (43)	0.192	0.133	1630 (51)	0.036*	0.573
**Mother (working status)**						
Full time	1033 (72)	0.002**	0.635	2076 (65)	0.261	0.126
Part time (0-50%)	100 (7)	0.396	0.211	383 (12)	0.037*	0.463
Part time (51-90%)	302 (21)	<.001***	0.418	736 (23)	0.002**	0.719
**Father (working status)**						
Full time	1076 (75)	0.016*	0.295	2651 (83)	0.101	0.154
Part time (0-50%)	86 (6)	0.105	0.103	159 (5)	0.199	0.163
Part time (51-90%)	273 (19)	0.088	0.118	385 (12)	<.001***	0.444
**Child sleep quality**						
Good	1105 (77)	<.001***	0.165	2971 (93)	<.001***	0.816
Bad	330 (23)	0.394	0.783	224 (7)	0.136	0.153
**Child health perception**						
Good	1349 (94)	0.361	0.133	3067 (96)	0.002**	0.638
Bad	86 (6)	<.001***	0.411	128 (4)	0.283	0.145

Table (3e): At age 17–23–years (*N*= 8558). *(Red box) strong corelation in Q1; (Green box) strong corelation in Q2.*

Variables	Less Active Group (Q1)			Highly Active Group (Q2)		
	(*n*= 2653) (%)	*p* value	*r* value	(*n*= 5905) (%)	*p* value	*r* value
**Sex**						
Male	1299 (49)	<.001***	0.661	2657 (45)	<.001***	0.702
Female	1354 (51)	<.001***	0.604	3248 (55)	<.001***	0.685
**Sports participation**						
Yes	663 (25)	0.164	0.173	4842 (82)	<.001***	0.783
No	1990 (75)	0.196	0.154	1063 (18)	0.173	0.274
**Child lives with**						
Both parents	1353 (51)	0.161	0.201	3661 (62)	0.019*	0.749
Split custody	212 (8)	0.002**	0.597	354 (6)	0.136	0.177
Mother (single)	53 (2)	0.016*	0.733	118 (2)	0.126	0.265
Father (single)	80 (3)	0.029*	0.386	236 (4)	0.287	0.172
Other	955 (36)	0.079*	0.154	1536 (26)	0.002**	0.793
**Siblings**						
0	-	-	-	-	-	-
1	-	-	-	-	-	-
2	-	-	-	-	-	-
≥ 3	-	-	-	-	-	-
**Accommodation type**						
House	-	-	-	-	-	-
Apartment	-	-	-	-	-	-
Other	-	-	-	-	-	-
**Animal pet (dog)**						
Yes	-	-	-	-	-	-
No	-	-	-	-	-	-
**Mother (smoking)**						
Yes	424 (16)	0.017*	0.569	1062 (18)	0.251	0.211

No	2229 (84)	0.113	0.158	4843 (82)	0.078	0.243
**Father (smoking)**						
Yes	212 (8)	<.001***	0.497	295 (5)	0.174	0.151
No	2441 (92)	0.251	0.116	5610 (95)	0.352	0.174
**Mother education level**						
≤ 9	238 (9)	<.001***	0.791	885 (15)	0.284	0.264
> 9 and ≤ 12	1883 (71)	0.173	0.164	3483 (59)	0.002**	0.411
> 12	532 (20)	0.291	0.199	1537 (26)	<.001***	0.779
**Father (education level)**						
≤ 9	530 (20)	<.001***	0.797	1417 (24)	0.177	0.179
> 9 and ≤ 12	743 (28)	0.018*	0.164	1299 (22)	0.113	0.224
> 12	1380 (52)	0.316	0.119	3189 (54)	<.001***	0.884
**Mother (working status)**						
Full time	2281 (86)	<.001***	0.573	4664 (79)	0.174	0.111
Part time (0-50%)	238 (9)	0.194	0.169	708 (12)	0.002**	0.499
Part time (51-90%)	134 (5)	0.274	0.372	533 (9)	0.032*	0.291
**Father (working status)**						
Full time	2440 (92)	<.001***	0.484	5550 (94)	0.263	0.261
Part time (0-50%)	159 (6)	0.173	0.173	118 (2)	0.217	0.196
Part time (51-90%)	54 (2)	0.217	0.184	237 (4)	0.002**	0.482
**Child sleep quality**						
Good	2016 (76)	0.056	0.116	3897 (66)	0.045*	0.718
Bad	637 (24)	0.002**	0.479	2008 (34)	0.284	0.119
**Child health perception**						
Good	1485 (56)	0.175	0.153	4546 (77)	<.001***	0.597
Bad	1168 (44)	0.013*	0.442	1359 (23)	0.193	0.154
